# Lack of association between vitamin D insufficiency and clinical outcomes of patients with COVID-19 infection

**DOI:** 10.1186/s12879-021-06168-7

**Published:** 2021-05-18

**Authors:** Alireza Davoudi, Narges Najafi, Mohsen Aarabi, Atefeh Tayebi, Roja Nikaeen, Hamideh Izadyar, Zahra Salar, Leila Delavarian, Narges Vaseghi, Zahra Daftarian, Fatemeh Ahangarkani

**Affiliations:** 1 Antimicrobial Resistance Research Center, Communicable Diseases Institute, Mazandaran University of Medical Sciences, Sari, Iran; 2 Department of Family Medicine, School of Medicine, Mazandaran University of Medical Sciences, Sari, Iran; 3 Department of Biostatistics, Faculty of Medical Sciences, Tarbiat Modares University, Tehran, Iran; 4 Mazandaran University of Medical Science, Ramsar International Branch, Ramsar, Iran; 5 Department of Pathobiology, Science and Research Branch, Islamic Azad University, Tehran, Iran

**Keywords:** Vitamin D, SARS-CoV-2, COVID-19, Coronavirus, Sufficiency, Insufficiency

## Abstract

**Background:**

A protective effect of vitamin D against COVID-19 infection is under investigation. We aimed to analyze the effect of vitamin D sufficiency on the clinical outcomes of patients infected with COVID-19.

**Methods:**

In this cross-sectional study we analyzed the vitamin D levels of COVID-19 patients who were admitted to Razi Hospital (an infectious disease referral center in Mazandaran province in northern Iran) from February to March 2020. Overall, a cutoff point of 30 ng/mL was used for the definition of vitamin D sufficiency.

**Results:**

One hundred fifty-three patients were analyzed in this study who had laboratory documentation of a 25(OH) D level at the time of hospitalization. The vitamin D levels of the patients were 27.19 ± 20.17 ng/mL. In total, 62.7% (*n* = 96) of the patients had a 25(OH) D level of less than 30 ng/mL and 37.25% (*n* = 57) had a 25(OH) D level of more than 30 ng/mL. In total, 49% (*n* = 75) of the patients suffered from at least one underlying disease. The univariate and multivariable regression showed that vitamin D sufficiency was not associated with a statistically significant lower risk of adverse clinical outcomes of COVID-19 such as duration of hospitalization and severity of infection (*P* > 0.05).

**Conclusions:**

Sufficient vitamin D levels were not found to be protective against adverse clinical outcomes in patients infected with COVID-19. Chronic disorders in COVID-19 patients were found to have greater relevance than vitamin D levels in determining the adverse outcomes of the infection. Further studies are needed to determine the role of vitamin D level in predicting the outcomes of COVID-19 infection.

## Introduction

Coronavirus 2019 or COVID-19, a respiratory infectious disease, has led to a pandemic of pneumonia-related illness [1–3]. The clinical features of this disease vary from asymptomatic or mild (in over 80%) to severe cases leading to acute respiratory syndrome and respiratory failure, requiring hospitalization in the intensive care unit, sepsis, septic shock and death [4–6]. The rapidly increasing number of infected individuals in highly critical states requiring intensive resources is a huge public health challenge worldwide [7]. Unfortunately, no suitable antiviral treatment has been found for this disease so far, and all therapeutics used are based on hypotheses that do not have sufficient evidence to support their use. Given the high economic importance of the COVID-19 pandemic, it is necessary to find methods that reduce the risk of infection and mortality at a low cost [8]. The status of the immune system is affected by multiple factors that may contribute to the risk of morbidity and mortality related to viral infections such as COVID-19. There are several vitamins and essential elements that have been shown to be necessary for a robust immune system response [9, 10]. Recent studies have highlighted a crucial supportive role for vitamin D in immune system functions, particularly in balancing the inflammatory response to viral infection [9, 11, 12]. Vitamin D is a fat-soluble vitamin that plays a key role in modulating both innate and adaptive immune responses. Previous studies have also shown that adequate levels of vitamin D have been shown to reduce the risk of viral respiratory infections and the length of hospital stay [1, 13, 14]. On the other hand, there is no accurate information about the COVID-19 virus in this field and the role of vitamin D supplementation in reducing the risk of infection is still under evaluation [15–17]. There is a high prevalence of vitamin D deficiency among Iranians based on several studies [18, 19]. Mazandaran province is located in the north of Iran, along the southern coast of the Caspian Sea with a moderate climate. In this study, we investigated the effect of vitamin D sufficiency on the clinical outcomes of patients with COVID-19 infection hospitalized in Razi hospital, which is a referral center of COVID-19 patients in Mazandaran province.

## Materials & methods

### Study design, participants and data source

This was a cross-sectional, retrospective study analyzing vitamin D levels of COVID-19 patients > 18 years old who were admitted to Razi Hospital, an infectious disease referral center in Mazandaran province, located in the north of Iran, from February to March 2020. The census method was used for sampling. The exclusion criteria was vitamin D intake in the past month prior to the diagnosis of COVID-19. The study was approved by the Ethics Committee of Mazandaran University of Medical Science, Ramsar international branch (IR.MAZUMS.RIB.REC.1399.019). Hospital medical records were analyzed from the inpatient database of Razi teaching hospital affiliated with Mazandaran University of Medical Sciences.

### Definitions and data collection

The definitive diagnosis of COVID-19 infection was based on the positive results of real-time reverse transcriptase-PCR (RT-PCR) assay for severe acute respiratory syndrome coronavirus-2 (SARS-CoV-2) using nasopharyngeal swab.

The vitamin D levels were measured by a high performance liquid chromatography (HPLC) method at the time of admission to the hospital. Based on the Endocrine Society’s Practice Guidelines on Vitamin D, overall, a cutoff point of 30 ng/mL was used for the definition of vitamin D sufficiency [20].

Definitions of COVID-19 severity were based on the World Health Organization COVID-19 clinical management guideline: 1- Mild stage (definitive COVID-19 patients without evidence of viral pneumonia or hypoxia); 2- Moderate disease (clinical signs of pneumonia [fever, cough, dyspnea, tachypnea] but no signs of severe pneumonia, including SpO2 ≥ 90% on room air not requiring supplemental oxygen); 3- Severe stage (with clinical signs of pneumonia [fever, cough, dyspnea, tachypnea) plus one of the following: respiratory rate > 30 breaths/min; severe respiratory distress; or SpO2 < 90% on room air; 4- Critical (Acute respiratory distress syndrome (ARDS), sepsis/septic shock, acute thrombosis) or other conditions that would normally require the provision of life sustaining therapies such as mechanical ventilation (invasive or non-invasive) or vasopressor therapy [21]. Severe and critical categories were grouped together as “severe” in our study.

Data such as demographic information (age, sex, body mass index [BMI], and living area), medical history, comorbidities, and diagnostic data of the patients, including vitamin D levels and the outcomes were extracted from medical files. Age was categorized into three classifications (50>, 50–65, and > 65 years old) [22]. The following outcomes were measured: duration of hospitalization, lung involvement, ICU admission, invasive and non-invasive mechanical ventilator use, severity of disease, and in-hospital mortality.

### Statistical analysis

Statistical analysis was performed using the Stata software version 14.0. Differences between groups (patients with 25(OH) D level ≥ 30 and patients with 25(OH) D level ≤ 30) were determined by the Chi-square test or Fisher’s exact test. Ordinal logistic regression was used to determine the association between variables and severity of diseases. Univariate linear regression was performed to evaluate the association between vitamin D status, demographic characteristics and existing comorbidities with adverse outcomes of COVID-19. Variables with a *P*-*value* less than 0.2 according to a univariate analysis and vitamin D status were included in the multivariable analysis. The descriptive values below 5% (*P*-value< 0.05) were considered statistically significant.

## Results

### Patient features and vitamin D status

A total of 841 patients with COVID-19 were admitted to Razi Hospital from February 2 to March 20, 2020, of which 153 patients were analyzed in this study who had laboratory documentation of a 25(OH) D level at the time of hospitalization. All patients had CT imaging findings typical of COVID-19 and a SARS-CoV-2 PCR-positive result. Median (range) vitamin D levels of the patients were 22.8 (3.2–89) ng/mL. In total, 62.7% (*n* = 96) of the patients had a 25(OH) D level of less than 30 ng/mL and 37.25% (*n* = 57) had a 25(OH) D level of more than 30 ng/mL. To assess the role of vitamin D status in relation to the disease, socio-demographic features, comorbidity factors, and clinical outcomes, all data were classified into two subgroups based on 25(OH) D levels. The socio-demographics and comorbidities and clinical outcomes of COVID-19 patients are presented in Tables 1 and 2.

**Table 1:** Characteristics of COVID-19 Patients Based on Vitamin D Level

	Total N (%)	25(OH) D level ≥ 30 ng/mL N (%)	25(OH) D level ≤ 30 ng/mL N (%)	***P***- ***value***
**Socio-demographic characteristics**				
**Gender**				
**Female**	66 (43.13)	23 (40.35)	43 (44.79)	0.601
**Male**	87 (53.86)	34 (59.64)	53 (55.2)	
**Age group (years old)**				
**50>**	55 (35.9)	13 (22.8)	42 (43.75)	**0.020**
**50–65**	54 (35.3)	22 (38.59)	32 (33.33)	
**> 65**	44 (28.8)	22 (38.59)	22 (22.91)	
**Body mass index (BMI)**	29.909 ± 5.81	29.15 ± 5.71	29.92 ± 5.92	0.321
**Living area**				
**Rural**	57 (37.25)	20 (35.08)	37 (38.54)	0.732
**Urban**	96 (62.7)	37 (64.91)	59 (61.45)	
**Preexisting comorbidities**				
**Diabetes**	41 (26.79)	19 (38.59)	22 (22.91)	0.183
**Hypertension**	44 (28.75)	26 (45.61)	18 (18.75)	**0.001**
**Cardiovascular disease**	30 (19.6)	17 (29.82)	13 (13.54)	**0.022**
**Dyslipidemia**	10 (6.53)	1 (1.75)	9 (9.37)	0.091
**Hypothyroidism**	9 (5.88)	0	9 (9.37)	**0.023**
**Asthma**	6 (3.92)	0	6 (6.25)	0.081
**Malignancy**	3 (1.96)	2	1 (1.04)	0.550
**Chronic liver disease**	2 (1.3)	1 (1.75)	1 (1.04)	1
**Chronic Kidney Disease**	1 (0.65)	1 (1.75)	0	0.371

*P-values* < 0.05 showed by boldface

**Table 2:** Clinical Outcomes of Patients infected with COVID-19 Based on Vitamin D Level

Outcomes of patients	Total N (%)	25(OH) D level ≥ 30 N (%)	25(OH) D level ≤ 30 N (%)	***P***- ***value***
**Bilateral lung involvement**	28 (17.64)	10 (17.54)	18 (17.7)	0.302
**ICU admission**	10 (6.53)	3 (5.26)	7 (7.29)	0.512
**Invasive mechanical ventilator use**	3 (1.96)	1 (1.75)	2 (2.083)	1
**Non-invasive ventilation**	15 (9.08)	5 (8.77)	10 (10.41)	1
**Mild form of COVID-19**	72 (41.7)	24 (42.1)	48 (50)	0.093
**Moderate form of COVID-19**	63 (41.2)	24 (42.1)	39 (40.62)	
**Severe form of COVID-19**	18 (11.8)	9 (15.78)	9 (9.37)	
**Death**	5 (3.26)	2 (3.57)	3 (3.12)	1
**Duration of hospitalization (days)**: **Mean ± SD**	6.3 ± 4.12	6.36 ± 4.35	6.25 ± 4.01	0.801

Based on the age group patients were distributed into three groups where 35.94% (*n* = 55) were less 50 years old, 34.64% (*n* = 53) were in the range of 50–65 years old, and 28.75% (*n* = 44) were over 65 years old respectively. 58.2% (*n* = 89) of patients were male, and 41.8% (*n* = 64) were female. There were no differences between the two groups (patients with 25(OH) D level ≥ 30 and patients with 25(OH) D level ≤ 30) in regards to gender and place of resident. Most of the patients had multiple underlying disorders. The most common underlying illnesses were hypertension 26.8% (*n* = 44), diabetes 26.8% (*n* = 41), cardiovascular disease 19.6% (*n* = 30), dyslipidemia 6.5% (*n* = 10), hypothyroidism 5.9% (*n* = 9), asthma 5.9% (*n* = 9), malignancy 1.96% (*n* = 3) and chronic liver disease 1.3% (*n* = 2), respectively. In total, 49% (*n* = 75) of patients suffered from at least one underlying disease. Significant differences between the two groups were noted to be preexisting comorbidities such as hypertension, cardiovascular disease, and hypothyroidism (*p* < 0.05). Hypertension (17/30) and cardiovascular disease (13/30) were mostly noted among patients with 25(OH) D level ≥ 30 ng/mL, while hypothyroidism was only seen in patients with 25(OH) D level ≤ 30 ng/mL.

The average hospitalization stay of patients were 6.3 ± 4.12 days. Bilateral lung involvement was seen in 17.64% of patients. In total, 6.53% of patients were admitted to the ICU. Invasive mechanical ventilator was utilized for 1.96% of patients while 9.08% of patients were under non-invasive ventilation. Based on severity of disease, 41.7% of patients were categorized into the mild form of COVID-19 disease, while 41.2 and 11.8% experienced the moderate and severe / critical forms of COVID-19, respectively. Overall, 3.26% of patients involved expired. Vitamin D sufficiency was not associated with a statistically significant lower risk of adverse clinical outcomes of COVID-19 such as duration of hospitalization, lung involvement, ICU admission, invasive and non-invasive ventilation, severity of disease, or death.

### Ordinal logistic regression analyses for severity of COVID-19

Ordinal logistic regression on parameters such as vitamin D level, gender, BMI, place of residence and preexisting comorbidities showed that patients with diabetes (OR 4, 95% CI (1.403–12.073%), *P* = 0.010), male patients (OR 3, 95% CI (1.413–6.056), *P* = 0.004) and patients living in rural areas (OR 2.5, 95% CI(1.295–5.025), *P* = 0.007) experienced the severe form of COVID-19 infection more often than other patients. However, there was no significant association between the other evaluated parameters and COVID-19 severity.

### Univariate and multivariable regression analysis for adverse outcome of COVID-19

There were few patients with some adverse outcomes of COVID-19 such as ICU admission (10/153), mortality (5/153), and invasive ventilation (3/153). Therefore, the univariate and multivariable linear regression was performed for hospitalization duration. The univariate and multivariable linear regression results showed that there was no significant association between vitamin D status and duration of hospitalization as well as the demographic variables and chronic disorders (Table 3).

**Table 3:** The Univariate and Multivariable Linear Regression Analysis of COVID- 19 Patients for Hospitalization Days

	Univariate Regression		Multivariable Regression	
Outcome	Duration of hospitalization (days)		Duration of hospitalization (days)	
Variables				
	Beta^a^	*P*-value	Beta^a^	*P*-value
**Vitamin D level**	0.01	0.876	−0.05	0.543
**Gender**	0.08	0.309	NA	NA
**Body mass index (BMI)**	−0.01	0.889	NA	NA
**Living Area**	−0.08	0.336	NA	NA
**Diabetes**	0.05	0.518	NA	NA
**Hypertension**	0.10	0.199	0.09	0.300
**Cardiovascular disease**	0.02	0.769	NA	NA
**Dyslipidemia**	−0.12	0.133	− 0.11	0.162
**Hypothyroidism**	−0.13	0.120	−0.12	0.159
**Asthma**	− 0.08	0.277	NA	NA
**Malignancy**	−0.03	0.683	NA	NA
**Chronic liver disease**	−0.02	0.784	NA	NA
**Chronic Kidney Disease**	−0.01	0.942	NA	NA

*NA* Not Applicable

^a^Coefficient Regression

## Discussion

The association between vitamin D status and seasonal respiratory infections has been proven in several studies [12, 23, 24]. Optimizing vitamin D status could improve the immune response and has been suggested as possibly protective in COVID-19 infection [10, 25, 26]. The COVID-19 outbreak began during the winter and a common feature of the inhabitants of all countries north of the 42nd parallel is a vitamin D insufficiency [27]. These facts resulted in the concept of using vitamin D for the prevention of COVID-19 infection or using vitamin D as an intervention strategy in COVID-19 patients [3, 23]. In our study, only 37.25% of patients had a sufficient vitamin D level. However, after categorizing all patients based on a cutoff point of 30 ng/mL for 25(OH) D to assess the association between vitamin D sufficiency and severity of COVID-19 infection, we found a lack of effect of vitamin D insufficiency on the clinical outcomes in patients with COVID-19 infection. Studies investigating the association of circulating vitamin 25(OH) D level and incidence and severity of COVID-19 are currently limited and prospective studies published to date are conflicting. Maghbooli et al. reported vitamin D sufficiency reduced the risk for adverse clinical outcomes in patients with COVID-19 infection, and Panagiotou1 et al. reported low vitamin D levels in patients hospitalized with COVID-19 are associated with greater disease severity [13, 14]. However, Azadeh et al. showed that vitamin D levels were lower in 80 patients with COVID-19 compared to 70 healthy individuals, while Brandão et al. reported there were no differences in the distribution of vitamin D levels between 2345 adults who were positive for SARS CoV-2 by RT-PCR test, and 11,585 negative controls [28, 29]. Consistent with our study findings, the study by Hastie et al. did not support a potential link between vitamin D concentrations and risk of COVID-19 infection [25, 28]. Older age and co-morbidities are linked to an insufficient vitamin D supply [30, 31]. It is notable that 64.05% of our patients were greater than 50 years old. Therefore, the high rate of patients in our study with vitamin D level ≤ 30 ng/mL may be due to the aging population. Unlike our study, where the severity of COVID-19 infection was not associated with lower vitamin D levels, Baktash et al. assessed the potential relationship between vitamin D deficiency and COVID-19 severity in hospitalized older adults and found that older patients with lower concentrations of vitamin D, when compared with age-matched vitamin D-replete patients, may demonstrate worse outcomes from COVID-19 [30]. Moreover, a meta-analysis by Zhao et al. on 53,000 COVID-19 patients, found that co-morbidities and old age showed a relationship with the renin angiotensin-aldosterone-system, vitamin D status, and COVID-19 infection [32]. After adjusting for socio-demographic features, comorbidity variables, and vitamin D level, ordinal logistic regression analyses results have shown that having diabetes, male gender, and residing in a rural area increased the risk of having the severe form of the COVID-19. Other studies have shown an increased risk of hospitalization and severe conditions requiring ventilation in patients with diabetes infected with COVID-19 [25, 33, 34]. In addition, demographic data reviewed from several studies highlighted the severity of COVID-19 was more significant in male compared to female patients [35, 36]. Moreover, delays in hospitalization and less access to health facilities can be factors involved in more severe forms of the disease among patients residing in rural areas.

Several studies have reported that a higher prevalence of vitamin D deficiency was observed in patients with worse COVID-19 outcomes [13, 14, 17, 37]. In our study, there was no evidence to suggest that assessment of vitamin D can serve as an indicator of the outcomes of COVID-19 infection. In line with our findings, Hastie et al. reported that the measurement of vitamin D would not be useful to evaluate the risk of COVID-19 in clinical practice [25].

There are some limitations in this research due to its cross-sectional retrospective design. The first drawback is that we included only patients who had documented vitamin D levels. The other drawback of our study is its small sample size, as well as some confounding factors, such as smoking and socioeconomic status were not recorded for most patients and could have an admissible impact on the COVID-19 severity.

## Conclusion

Despite the fact that the number of patients with an insufficient vitamin D level was greater in our study, and approximately 44% of patients fell into the moderate and severe/critical categories, some requiring invasive/non-invasive ventilation, ICU admission, longer hospitalization, and death, there was no statically significant evidence that insufficient vitamin D levels might play a role in adverse outcomes of COVID-19 infection.

Chronic disorders in COVID-19 patients likely have greater relevance than vitamin D levels in determining the adverse outcomes of the infection. Therefore, vitamin D supplementation may not be helpful in the prevention or treatment of COVID-19. As there is still no effective pharmaceutical treatment for COVID-19, general vaccination of the community, as well as adherence to health protocols, are the main tools to reduce morbidity and mortality caused by this disease. Larger prospective studies are needed however to support or refute our study findings.

## Data Availability

All data analyzed during this study are included in this published article.

## Abbreviations

OR: Odds ratio
CI: Confidence interval
SPO2: Peripheral oxygen saturation
BMI: Body mass index
ICU: Intensive care unit
Not Applicable: NA
